# A Case of Pneumoscrotum Following Spontaneous Colonic Perforation and Mimicking Strangulated Inguinal Hernia

**Published:** 2014-01-31

**Authors:** Rahele Mehraeen, Soheil Osia

**Affiliations:** 1 Department of Radiology; 2 Department of Surgery, Amirkola Hospital, Babol University of Medical Sciences, Babol, Iran

**Keywords:** Pneumoscrotum, Perforation, Inguinal Hernia

**Sir,**

We would like to present an idiopathic sigmoid colon perforation revealed with a rare manifestation of pneumoscrotum.

**Fig. 1:**
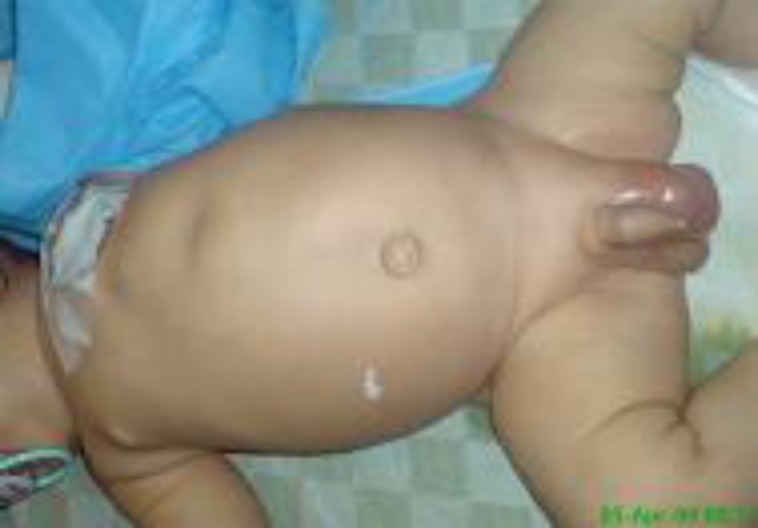
A 42 day old boy with agitation, progressive abdominal distention and an erythematous swollen scrotum

 A forty two days old boy was referred to us because of agitation, progressive abdominal distention and swollen scrotum (Fig. 1). There were not any symptoms of other gastrointestinal tract such as nausea, vomiting, diarrhea, fever and the change of the stool color.

 On physical examination, the infant had tachycardia and tachypnea. His abdomen had distention but was soft and lax, and his scrotum had swelling with red skin that led us into a consideration of strangulated inguinal hernia.

**Fig. 2:**
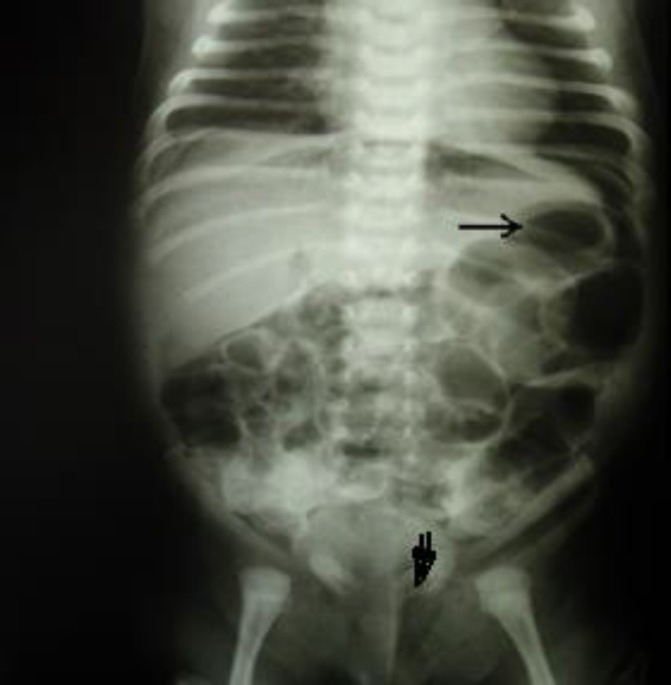
Abdominal x-ray shows free intraperitoneal gas and Rigler sign (gas in the inner and outer side of the bowel loop- arrow).The open arrow shows gas in the left scrotal sac.

 Portable CXR was normal but plain abdominal x-ray three hours later revealed bowel distention, specially in the distal colon, with free intra-peritoneal gas (Rigler's sign)(Fig 2). A small bubble of gas was seen in the left scrotal region. According to the Rigler's sign, we made a tentative diagnosis of bowel perforation resulting from left sided inguinal hernia.

 An urgent ultrasound of the scrotum revealed only reverberation artefact, consistent with gas. Both testes were normal. An exploratory laparotomy was done through midline incision. The peritoneum was grossly contaminated and there was perforation of the descending and sigmoid colon on the antimesenteric side . Rest of the large and small bowel was normal and there were not any vesiculation or ulceration and the mucosal surfaces were intact.

 The mucosal biopsy specimen from the edge of perforation had a normal appearance under light microscopy, and there were not any characteristic CMV inclusions or HSV cytopathic changes. However, we had not immunohistochemistry, FISH, and PCR in our center to detect latent infection. Also multiple distal biopsies revealed normal ganglion cells and Hirschprung's disease was excluded.

 The incidence of spontaneous colonic perforation is extremely rare. As in one study, it was reported only ten cases, seven of ten in very-low-birth-weight (VLBW) infants and three of ten in term or near-term neonates (one with Hirschsprung disease and the two cases without clear etiology during 10 years (2000-2009)[1].

 Prompt diagnosis and early vigorous management are mandatory for neonatal colonic perforation, because of the highest mortality rate of it among neonatal gastrointestinal perforations which was reported in some studies[2].

 Since 1912 (the first reported case)^[^^3^^]^, there are few reported pneumoscrotum due to procedural or pathologic processes^[^^4^^]^. We think that our patient was the first case which reported spontaneous colonic perforation with unknown etiology, which presented with pneumoscrotum during the last decades.

 Endoscopic (upper and lower) and laparoscopic procedures were the second and third most common causes, respectively^ [^^5^^]^.

 Therefore, as mentioned above, the finding of gas in the scrotal sac may be an early sign of a life-threatening condition like visceral perforation or may represent an incidental finding associated with more benign conditions like laparoscopic procedures.

 In conclusion, although a combination of free abdominal gas and distended abdomen usually make the diagnosis evident and surgical intervention should be the rule^[9]^, this case presentation reminds us that eventhough pneumoscrotum is a benign, rare condition, its mere presence should signal the possibility of a severe, life-threatening disease process within the peritoneum like bowel perforation or retroperitoneum like pancreatic or renal abscess. 
